# Quality Characteristics and Acceptance Intention for Healthcare Kiosks: Perception of Elders from South Korea Based on the Extended Technology Acceptance Model

**DOI:** 10.3390/ijerph192416485

**Published:** 2022-12-08

**Authors:** Uk Kim, Taerin Chung, Eunsik Park

**Affiliations:** 1 Department of Life Sports, Dankook University, 119, Dandae-ro, Cheonan-si 31116, Chungcheongnam-do, Republic of Korea; 2 Deparment of Life Sports Education, Kongju National University, Gongjudaehak-ro 56, Gongju-si 32558, Chungcheongnam-do, Republic of Korea

**Keywords:** path analysis, healthcare kiosk, quality characteristics, extended technology acceptance model, elderly

## Abstract

This study aimed to perform a path analysis to understand the effects of quality characteristics on perceived usefulness, perceived ease to use, involvement, and acceptance intention of healthcare kiosks in elderly using the extended technology acceptance model. We performed structural equation modeling (SEM) with data from 300 elderly. The following results were obtained. Firstly, elderly’s perceived quality characteristics of healthcare kiosks had a partial positive effect on perceived usefulness. Secondly, elderly’s perceived quality characteristics of healthcare kiosks had a partial positive effect on perceived ease to use. Thirdly, elderly’s perceived ease to use healthcare kiosks had a partial positive effect on perceived usefulness. In addition, elderly’s perceived usefulness of healthcare kiosks had a positive effect on acceptance intention. Lastly, elderly’s perceived ease to use healthcare kiosks had a positive effect on acceptance intention.

## 1. Introduction

### 1.1. Introduction

**Figure 1:**
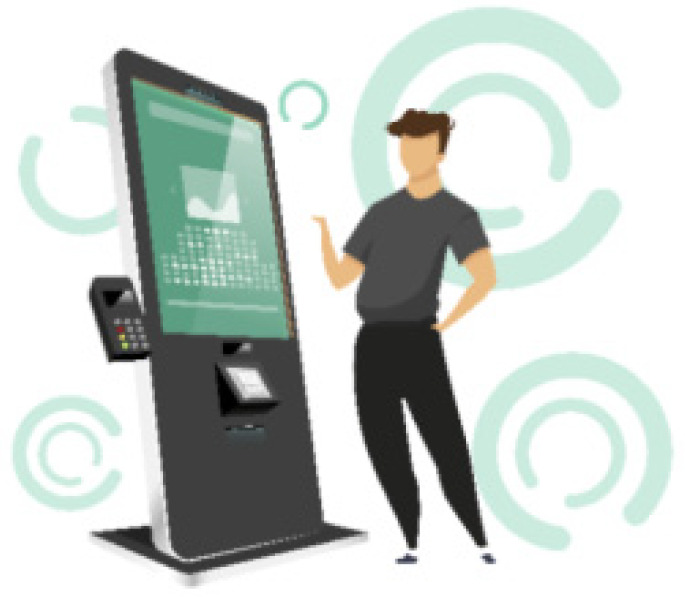
Image of a Healthcare Kiosk [9].

“Healthcare Device” is a collective term for digitalized equipment used for healthcare, such as watches, scales, and kiosks [1,2]. Of the healthcare devices, healthcare kiosks have been highlighted as a promising in modern society with high potential as a useful technology for elderly who have difficulty with self-care [3,4,5]. Healthcare kiosks specifically refer to rectangular shaped touch screens that are equipped with motion recognition, voice recognition sensors and automatic intelligence, etc. [6] (Figure 1). Healthcare kiosks are also equipped with software. The software enables measurement and analysis of people’s health-related data in real time, so users can manage their own health at home or primary residence without having to visit a hospital or health management center in person. The software installed in healthcare kiosks is equipped with functions that can measure body composition, blood pressure, take electrocardiograms, and cholesterol levels, and physical abilities of the elderly, such as muscle strength, muscular endurance, and cardiorespiratory endurance etc. The first commercialized form of a healthcare kiosk was a wearable device resembling a watch, but its high cost significantly limited its availability [7,8].

In recent years, user-friendly devices that can be used in public places have been developed. The innovative advances in healthcare kiosks are attributable to the fact that the technology promotes active interdisciplinary integration, allowing the creation of a new pioneering market with high profit and diverse opportunities [10,11]. Therefore, this technology is expected to play a pivotal role in driving national growth and enterprise in Korea, a country with one of the highest levels of aging. However, the problem is that it is very challenging to achieve the goal of promoting self-management using kiosks, which are often perceived as being a difficult skill for the elderly, where health care is essential in their daily life [12,13]. In Korea, various forms of kiosks have been developed and implemented in places such as banks, fast food restaurants, airports, and terminals. However, there are concerns about elderly choosing to avoid such novel technology developed to enhance convenience due to usage difficulty.

Therefore, it is essential to examine the elderly’s acceptance of healthcare kiosks. The Extended Technology Acceptance Model (ETAM) is understood as the most useful study model for identifying the usability of innovative technology [14]. The ETAM is a modified version (deletion of Attitude toward using) of the Technology Acceptance Model (TAM) introduced by [15], and the primary components are perceived ease to use, perceived usefulness, and acceptance intention.

### 1.2. Purpose of Study

This study aimed to analyze the causality between quality characteristics of healthcare kiosks and perceived usefulness, perceived ease of use, and acceptance intention among elderly through a path analysis.

### 1.3. Hypotheses

**Figure 2:**
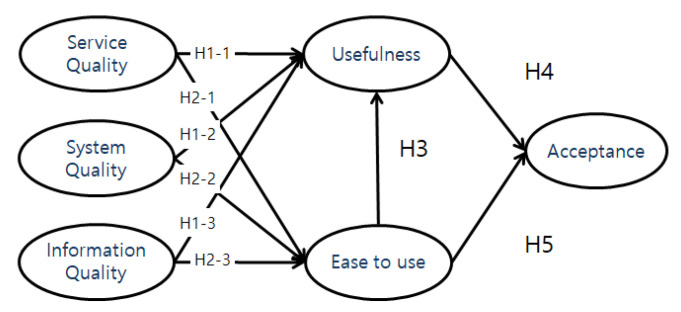
Research Model.

The following hypotheses were established to this end (Figure 2):

**H1-1.**  *Elderly’s perceived service quality of healthcare kiosks will have a positive effect on perceived usefulness of healthcare kiosks*.

**H1-2.**  *Elderly’s perceived system quality of healthcare kiosks will have a positive effect on perceived usefulness of healthcare kiosks.*

**H1-3.**  *Elderly’s perceived information quality of healthcare kiosks will have a positive effect on perceived usefulness of healthcare kiosks*.

**H2-1.**  *Elderly’s perceived service quality of healthcare kiosks will have a positive effect on perceived ease to use healthcare kiosks*.

**H2-2.**  *Elderly’s perceived system quality of healthcare kiosks will have a positive effect on perceived ease to use healthcare kiosks*.

**H2-3.**  *Elderly’s perceived information quality of healthcare kiosks will have a positive effect on perceived ease to use healthcare kiosks*.

**H3.**  *Elderly’s perceived ease to use of healthcare kiosks will have a positive effect on perceived usefulness of healthcare kiosks*.

**H4.**  *Elderly’s perceived usefulness of healthcare kiosks will have a positive effect on acceptance intention*.

**H5.**  *Elderly’s perceived ease to use healthcare kiosks will have a positive effect on acceptance intention*.

## 2. Research Methods

### 2.1. Research Subjects

To achieve the objective of this study, online and offline questionnaire surveys were administered to panels of 300 elderly participating in the development of a healthcare kiosk. A sample was derived via convenience sampling from the population, and the surveys were administered in person or via the internet. Considering the ongoing COVID-19 pandemic, the first 300 questionnaires were administered via Google Survey at five senior welfare facilities and long-term care hospitals in three cities of the Republic of Korea, and all questionnaires were retrieved. Specifically, 100 paper-based questionnaires and 200 online-based survey links were administered at two senior welfare centers in Seoul and Seongnam, Gyeonggi Province, respectively, and all questionnaires were retrieved. From the collected questionnaires, 31 inappropriate surveys such as incomplete surveys, answering wrong numbers, missing questions, careless responses like returning the paper very quickly for rewards, were excluded. A total of 269 questionnaires were included in the analysis. Table 1 shows the demographic characteristics of the participants.

### 2.2. Research Tools

A questionnaire was used as the survey method. Table 2 contains the summarized questionnaire scale.

### 2.3. Analysis Procedure

The collected data were analyzed using SPSS 230.0 and AMOS 210.0. Specifically, SPSS 230.0 was used for the frequency analysis to analyze demographic characteristics, and reliability and correlation analysis were performed to test the questionnaire’s reliability and multicollinearity, respectively. AMOS 21.0 was used for confirmatory factor analysis (CFA) to establish the validity of the latent variables to test the validity between factors, the causal relationships between variables, and the hypothesis. After checking the parameter estimates and GOF (Goodness of Fit) of the measurement model based on the values of the CFI (comparative fit index), TLI (Tucker–Lewis Index), and RMSEA (Root Mean Square Error of Approximation), the validity between the observation and latent variables was tested. Lastly, hypothesis were accepted or refused by testing the causal relationships between the variables for the structural modeling analysis.

### 2.4. The Validity and Reliability of the Measured Variables

The internal validity of each study variable was assessed by the CFA. During this process, four items (two items in service quality, one in easy to use, and one in usefulness) were determined to diminish internal validity and thus were excluded. The results are shown in Table 3.

The internal validity results for each variable were applied to the overall model, and the discriminant validity and reliability of each variable were evaluated. Discriminant validity can be evaluated based on construct reliability (CR) and average variance explained (AVE), where discriminant validity is considered established with ≥0.500 and 0.700, respectively. Reliability is evaluated using Cronbach’s α. In general, a Cronbach’s α of 0.700 or higher is considered to indicate good reliability. The CR, AVE, and Cronbach’s α values are shown in Table 4.

## 3. Results

### 3.1. The Results of Correlation Analysis

The relationships among elderly’s perceived quality characteristics of healthcare kiosks, ease of use, usefulness, and acceptance intention were analyzed with the Pearson correlation analysis. The results are shown in Table 5. The correlations among each variable were below 0.700, confirming the absence of multicollinearity.

### 3.2. Results of the Structural Modeling Analysis

To achieve the procedure and goals designed in this study, the fit of the overall model was analyzed. The fit indices were: χ^2^ of 479.153, *df* of 365, TLI of 0.964, CFI of 0.968, and RMSEA of 0.034. The results are shown in Table 6.

### 3.3. Results of Hypothesis Verification

**Figure 3:**
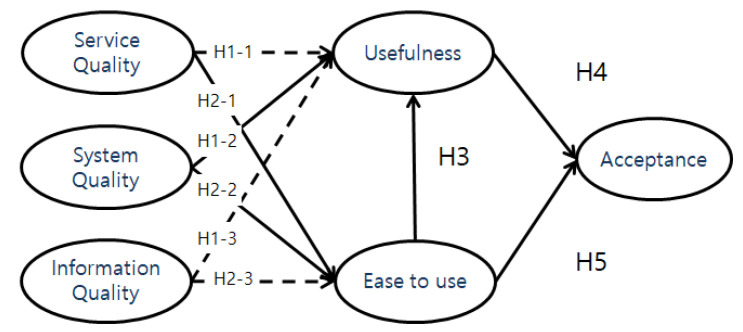
Research Results.

The results of hypothesis testing are shown below (Figure 3). H1-1, pertaining to the path from service quality of healthcare kiosks to perceived ease to use, was rejected (*t* = 1.351). H1-2, pertaining to the path from system quality of healthcare kiosks to perceived ease to use, was accepted (*t* = 2.814, *p* < 0.01). H1-3, pertaining to the path from information quality of healthcare kiosks to perceived ease to use, was rejected (*t* = 1.033). H2-1, pertaining to the path from service quality of healthcare kiosks to perceived usefulness, was accepted (*t* = 2.304, *p* < 0.05). H2-2, pertaining to the path from system quality of healthcare kiosks to perceived usefulness, was accepted (*t* = 3.362, *p* < 0.001). H2-3, pertaining to the path from information quality of healthcare kiosks to perceived usefulness, was rejected (*t* = −0.432). H3, pertaining to the path from perceived ease to use healthcare kiosks to perceived usefulness, was accepted (*t* = 2.675, *p* < 0.01). H4, pertaining to the path from perceived usefulness of healthcare kiosks to acceptance intention, was accepted (*t* = 3.830, *p* < 0.001). H5, pertaining to the path from perceived ease to use healthcare kiosks to acceptance intention, was accepted (*t* = 2.014, *p* < 0.05). These results are delineated in Table 7.

## 4. Discussion

Some points of discussion, drawn up according to the results of the study are summarized.

**Figure 4:**
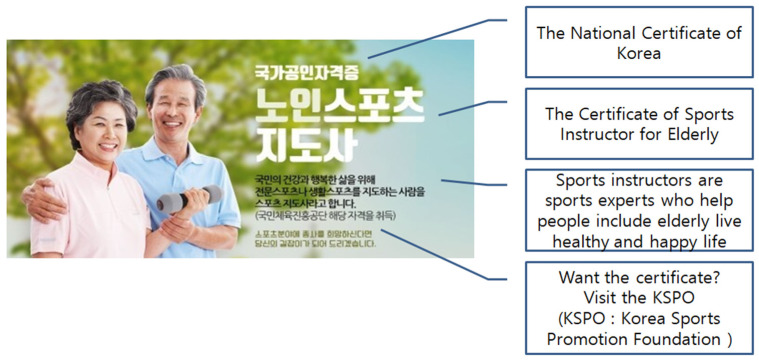
Recruiting Advertisement for Sports Instructor Certificate for the Elderly in Korea.

First, elderly demonstrated intention to accept healthcare kiosks if they perceived the kiosk services as easy to use. These kiosks, including the software and its services, can be defined as adding structure and technology to elderly’s perceived services to use kiosks and enhances their understanding and expectations [18,19]. In Korean culture, the ease to use and understand kiosk services for the elderly is understood as being able to obtain the desired services with the help of an assistant [20,21]. This is the reason why the Korean government issues a certificate for guiding physical activities and sports in the elderly in Korea with the title of “National Sports Instructor for the Elderly of Korea” (Figure 4). In recent years, the expansion of service industries, such as the functioning of the device for an auxiliary role or a manpower training business, for internationally, could be understood in the same context [22,23]. Thus, it can be said to have been proven that easily understood and were motivated to accept using healthcare kiosks in the presence of a service providing a helper to guide them through the technology. Therefore, elderly’s intentions in adopting the technology with the technological development of a health care device like health kiosk, should be improved to make health care more effective [24,25,26]. To accomplish this, fostering and training an assistive personnel industry and improving the accessibility of the industry that is supposed to be invested in boosting healthcare kiosks, is required.

**Figure 5:**
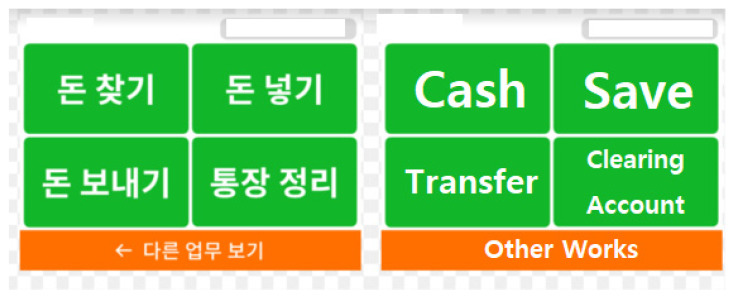
Image of ATM Kiosk Screen of a Bank in Korea.

Second, were motivated to accept healthcare kiosks if they perceived these to be easy to use and useful in their daily lives. Healthcare kiosks are state-of-the art machines that are aimed at promoting health management among elderly, as well as the entire human population, more effectively [2,3,27]. Therefore, the system is quickly being advanced and becoming more sophisticated [28]. Many scholars have already substantiated that user utilization declines if system quality deteriorates [29,30]. However, this applies only to the majority younger generations or very tiny number of elderly who can adapt to system advances easily. Specifically, it might be impossible for elderly to keep abreast of, and fully utilize, scientific advances of operating systems, and study findings support such hypothesis [31,32]. Hence, healthcare kiosks using systems that consider the level of cognitive and adaptive capacities of elderly must be developed. In this context, it would be more appropriate to develop basic and convenient devices, as opposed to highly advanced technologies using artificial intelligence or the meta-verse. A launch of an ATM service for at a bank in Korea is a good example (Figure 5).

Third, it is difficult to motivate to accept healthcare kiosks through their perceived information quality of kiosks. These results vividly demonstrate the challenges face in using these kiosks, which is an emerging societal issue [33,34]. Specifically, at the point of this research the results were interpreted as the elders remembering that the information provided by healthcare kiosks that they experienced were mostly difficult to understand and impractical to them. Thus, to inspire elderly to accept healthcare kiosks, they should be filled with information that is easy to understand and applicable in their daily lives, to instill a positive memory of kiosks [35,36].

### 4.1. Conclusions

Amid the super-aging of human societies across regions and cultures, health management among elderly holds significance in enhancing human quality of life. Thus, a variety of innovative devices have been developed to facilitate a scientific approach to human health management, and healthcare kiosks have been highlighted as an innovative technology to maximize the effectiveness of health management. However, there are doubts about the effectiveness of these healthcare kiosks. This is because physical and cognitive aging of humans hinder individuals to keep abreast with scientific advances, and this gap is widening. Moreover, healthcare kiosks equipped with more specialized systems, services, and information would be more challenging to use by who have difficulty utilizing more basic and convenient kiosks in airports, restaurants, and movie theaters.

Therefore, we determined that it is essential to investigate what features of healthcare kiosks would motivate to accept the technology, and we have achieved our goal to some degree. We hope that our findings serve as important data for developing health management devices not only for elderly but for the entire human race.

### 4.2. Limits of Study

This study has a few limitations.

First, the study participants only comprised aged 65 years and over. Thus, generalizing the findings to the entire age group may cause significant errors. Subsequent studies should expand the age group of the study population.

Second, the healthcare device of interest in this study was kiosks. Thus, the findings cannot be generalized to other smart devices, such as watches and tablets. Future studies could consider expanding the scope of healthcare devices.

Third, the questionnaire responses were dependent on elderly’s experiences with kiosks. Thus, the findings cannot be applied to other forms of kiosks or healthcare devices to be developed.

## Figures and Tables

**Table 1:** Demographic Analysis of Research Subjects.

Contents		Frequency	Percentages
Gender	Male	128	47.6
	Female	141	52.4
Ages	60~65	65	23.8
	66~70	44	16.7
	70~75	68	21.6
	76~80	42	15.6
	81~85	30	11.2
	86~90	12	4.5
	Higher than 90	18	6.7
Self Health Evaluation	Very Good	57	21.2
	Good	41	15.2
	I don’t know well	70	26.0
	A Bit Bad	43	16.0
	Very Bad	58	21.6
Education	Middle School	86	32.0
	High School	160	59.9
	College	12	4.5
	Grad School	11	4.1
Disability	Registered Disabled	34	12.6
	Not Disabled	219	81.4
	Nonregistered Disabled	16	6.0
Total		269	100.0

**Table 2:** Research Tools.

Variables	Sub-variables	# of Questions	References
Quality Characteristics	Service Quality	6	[16]
	System Quality	4	
	Information Quality	5	
Ease to Use		6	[14]
Usefulness		6	[14]
Acceptance Intention		6	[16,17]

**Table 3:** Results of Confirmative Factor Analysis II.

Variables		Early Qs	Final Qs	χ^2^	*df*	TLI	CFI	RMSEA
Quality Characteristics	Service Q	6	4	117.778	62	0.947	0.958	0.058
	System Q	4	4					
	Information Q	5	5					
Ease to Use		6	5	12.103	5	0.979	0.990	0.070
Usefulness		6	5	12.655	5	0.973	0.985	0.076
Acceptance Intention		6	6	17.591	9	0.980	0.988	0.060
Total		33	29	478.411	362	0.963	0.967	0.035

**Table 4:** Results of Confirmative Factor Analysis II and Reliability Analysis.

Variables	Contents	Est	SE	CR	AVE	α
Service Quality	1. The device runs fast.	0.768	0.302	0.540	0.824	0.819
	2. The connection speed is high.	0.708	0.296			
	3. The device has effective features.	0.719	0.333			
	6. It is easy to access information.	0.742	0.380			
System Quality	7. It responds quickly.	0.754	0.277	0.536	0.822	0.822
	8. It provides information relevant to me.	0.769	0.354			
	9. I can expect my desired service.	0.688	0.354			
	10. I think it will continue to provide the same services as now.	0.715	0.394			
Information Quality	11. Information is accurate.	0.713	0.305	0.523	0.845	0.812
	12. Information is clear.	0.761	0.389			
	13. Adequate information is provided.	0.735	0.311			
	14. Most up-to-date information is provided.	0.747	0.428			
	15. It is useful for obtaining information.	0.654	0.410			
Ease to Use	16. I can learn things more easily.	0.739	0.248	0.560	0.864	0.865
	17. It allows me to control things I wish to do.	0.725	0.230			
	18. I can do what I wish to do with more flexibility.	0.729	0.255			
	19. It allows to me to achieve what I want to do with skills.	0.764	0.292			
	21. I can learn things I want to learn more easily.	0.784	0.342			
Usefulness	22. I can do things I want to do more quickly.	0.775	0.304	0.565	0.866	0.864
	24. It makes good outcomes.	0.773	0.359			
	25. It is efficient.	0.773	0.358			
	26. It is effective.	0.735	0.322			
	27. It is useful for doing things I want to do.	0.700	0.292			
Acceptance Intention	28. I will use it again in the future.	0.737	0.320	0.551	0.860	0.881
	29. I will recommend it to others.	0.730	0.300			
	30. I will use this as my go-to device for learning.	0.736	0.275			
	31. I will accept the information provided	0.756	0.266			
	32. I will search the provided information.	0.751	0.303			
	33. I will recommend the provided information to others.	0.756	0.314			
χ^2^= 478.411, *df* = 362, TLI = 0.963, CFI = 0.967, RMSEA = 0.035						

**Table 5:** Results of Confirmative Factor analysis and Reliability Analysis.

	System	Service	Information	Usefulness	Ease	Acceptance
System Quality	1					
Service Quality	0.688 **	1				
Information Quality	0.133 *	0.104	1			
Usefulness	0.564 **	0.522 **	0.113	1		
Ease to Use	0.585 **	0.524 **	0.082	0.539 **	1	
Acceptance Intentions	0.326 **	0.248 **	0.088	0.371 **	0.323 **	1

* *p* < 0.05, ** *p* < 0.01.

**Table 6:** Fit of Entire Model.

Model #	χ^2^	*df*	TLI	CFI	RMSEA
1	479.153	365	0.964	0.968	0.034

**Table 7:** Results of Analysis on direct Effects.

H	Path			Est	SE	T	Results
1-1	Service Quality	→	Usefulness	0.146	0.108	1.351	Rejected
1-2	System Quality	→	Usefulness	0.357	0.127	2.814 **	Accepted
1-3	Information Quality	→	Usefulness	0.066	0.064	1.033	Rejected
2-1	Service Quality	→	Ease to Use	0.283	0.123	2.304 *	Accepted
2-2	System Quality	→	Ease to Use	0.465	0.138	3.362 ***	Accepted
2-3	Information Quality	→	Ease to Use	−0.031	0.03	−0.432	Rejected
3	Ease to Use	→	Usefulness	0.202	0.076	2.675 **	Accepted
4	Usefulness	→	Acceptance Intention	0.335	0.088	3.830 ***	Accepted
5	Easy to Use	→	Acceptance Intention	0.153	0.076	2.014 *	Accepted

* p < 0.05, ** *p* < 0.01, *** *p* < 0.001.

## Data Availability

Not applicable.

## References

[1] Nakata S., Arie T., Akita S., Takei K. (2017). Wearable, flexible, and multifunctional healthcare device with an ISFET chemical sensor for simultaneous sweat pH and skin temperature monitoring. ACS Sens..

[2] Yeh P.C. (2020). Impact of button position and touchscreen font size on healthcare device operation by older adults. Heliyon.

[3] Lee J.I., Park S.C., Yang S.C., Lee W.J. (2018). Design and implementation of personal health information system using smart health care zone. J. Adv. Inf. Technol. Converg..

[4] Barrett D.I., Kalnad N. (2014). Deployment of digital healthcare kiosks in the workplace: Utilisation and acceptability. Int. J. Integr. Care.

[5] Bagula M.F., Bagula H., Mandava M., Lubamba C.K., Bagula A. Cyber-healthcare kiosks for healthcare support in developing countries. Proceedings of the International Conference on e-Infrastructure and e-Services for Developing Countries.

[6] Tony R.A.J., Sarah T., Dhinagaran D., Ugargol A.P. (2013). Design, development and implementation of a touch-screen health information kiosk for patients at the outpatient waiting area in a large tertiary care hospital in India: An evaluation of user satisfaction. J. Health Inform. Dev. Ctries..

[7] Iqbal S., Mahgoub I., Du E., Leavitt M.A., Asghar W. (2021). Advances in healthcare wearable devices. NPJ Flex. Electron..

[8] Cima M.J. (2014). Next generation wearable electronics. Nat. Biotechnol..

[9] Rumi M.S. How Health Care Kiosk Is Changing the Medical Landscape. https://medium.com/@amisiga/how-health-care-kiosk-is-changing-the-medical-landscape-d51f68f85b01.

[10] Lillrank P. (2015). Learning from Industry: Innovating in Health-Care Operation.

[11] Sousa V.D., Rojjanasrirat W. (2011). Translation, adaptation and validation of instruments or scales for use in cross-cultural health care research: A clear and user-friendly guideline. J. Eval. Clin. Pract..

[12] Perry T.E., Ruggiano N., Shtompel N., Hassevoort L. (2015). Applying Erikson’s wisdom to self-management practices of older adults: Findings from two field studies. Res. Aging.

[13] Ahn H.S., Kuo I.H., Datta C., Stafford R., Kerse N., Peri K., Broadbent E., MacDonald B.A. (2014). Design of a Kiosk Type Healthcare Robot System for Older People in Private and Public Places. SIMPAR 2014: Simulation, Modeling, and Programming for Autonomous Robots.

[14] Davis F.D. (1989). Perceived usefulness, perceived ease of use, and user acceptance of information technology. MIS Q..

[15] Martín-García A.V., Redolat R., Pinazo-Hernandis S. (2022). Factors Influencing Intention to Technological Use in Older Adults. The TAM Model Aplication. Res. Aging.

[16] Luttenberger K., Reppermund S., Schmiedeberg-Sohn A., Book S., Graessel E. (2016). Validation of the Erlangen test of activities of daily living in persons with mild dementia or mild cognitive impairment (ETAM). BMC Geriatr..

[17] Dasgupta A., Sansgiry S.S., Sherer J.T., Wallace D., Sikri S. (2009). Application of the extended technology acceptance model in predicting pharmacists’ intention to use personal digital assistants. J. Am. Pharm. Assoc..

[18] Youm S., Park S.H. (2015). How the awareness of u-healthcare service and health conditions affect healthy lifestyle: An empirical analysis based on a u-healthcare service experience. Telemed. e-Health.

[19] Huntington P., Williams P., Nicholas D. (2002). Age and gender user differences of a touch-screen kiosk: A study of kiosk transaction log files. J. Innov. Health Inform..

[20] Choi J.S., Lee S. (2014). Service Quality Control for Nursing Homes in South Korea: Regulation vs. Evaluation?. Res. Seoul Inst..

[21] Choi D., Choi H., Shon D. (2019). Future changes to smart home based on AAL healthcare service. J. Asian Archit. Build. Eng..

[22] Kim K.S. (2020). A Research on the Methods for Advancement of LongTerm Care Workforce in Korea through Cases in Germany: Focusing on the Development in the Occupational System of Caregivers and in Candidate Training in Germany. Korean Acad. Qual. Res. Soc. Welf..

[23] Bookey-Bassett S., Markle-Reid M., Mckey C.A., Akhtar-Danesh N. (2017). Understanding interprofessional collaboration in the context of chronic disease management for older adults living in communities: A concept analysis. J. Adv. Nurs..

[24] Wang K.H., Chen G., Chen H.G. (2017). A model of technology adoption by older adults. Soc. Behav. Personal. Int. J..

[25] Pan S., Jordan-Marsh M. (2010). Internet use intention and adoption among Chinese older adults: From the expanded technology acceptance model perspective. Comput. Hum. Behav..

[26] Nikou S., Agahari W., Keijzer-Broers W., de Reuver M. (2020). Digital healthcare technology adoption by elderly: A capability approach model. Telemat. Inform..

[27] Van Der Krieke L., Wunderink L., Emerencia A.C., De Jonge P., Sytema S. (2014). E–mental health self-management for psychotic disorders: State of the art and future perspectives. Psychiatr. Serv..

[28] Ventola C.L. (2014). Mobile devices and apps for health care professionals: Uses and benefits. Pharm. Ther..

[29] Ng G., Tan N., Bahadin J., Shum E., Tan S.W. (2016). Development of an automated healthcare kiosk for the management of chronic disease patients in the primary care setting. J. Med. Syst..

[30] Lyu Y., Vincent C.J., Chen Y., Shi Y., Tang Y., Wang W., Ding J. (2015). Designing and optimizing a healthcare kiosk for the community. Appl. Ergon..

[31] Letafat-Nejad M., Ebrahimi P., Maleki M., Aryankhesal A. (2020). Utilization of integrated health kiosks: A systematic review. Med. J. Islam. Repub. Iran.

[32] Eisma R., Dickinson A., Goodman J., Mival O., Syme A., Tiwari L. (2003). Mutual inspiration in the development of new technology for older people. Proc. Incl..

[33] Rogers W., Fisk A. (2006). Cognitive support for elders through technology. Generations.

[34] Fischer S.H., David D., Crotty B.H., Dierks M., Safran C. (2014). Acceptance and use of health information technology by community-dwelling elders. Int. J. Med. Inform..

[35] Altizer K.P., Grzywacz J.G., Quandt S.A., Bell R., Arcury T.A. (2014). A qualitative analysis of how elders seek and disseminate health information. Gerontol. Geriatr. Educ..

[36] Caprani N., O’Connor N.E., Gurrin C. (2012). Touch screens for the older user. Assistive Technologies.

